# Unveiling the dual reactivity of nanoscaled PuO_2_ sonicated in oxygenated aqueous solutions^☆^

**DOI:** 10.1016/j.ultsonch.2025.107346

**Published:** 2025-04-08

**Authors:** Julien Margate, Matthieu Virot, Thomas Dumas, Simon Bayle, Denis Menut, Laura Bonato, Emilie Broussard, Fanny Molière, Charles Hours, Laurent Venault, Sergey I. Nikitenko

**Affiliations:** a ICSM, Univ Montpellier, CEA, CNRS, ENSCM, Marcoule, France; b CEA, DES, ISEC, DMRC, Univ Montpellier, Marcoule, France; c Synchrotron SOLEIL, L’Orme des Merisiers, Saint-Aubin, France

**Keywords:** Plutonium, Peroxide, Nanoparticle, Dissolution, Actinide

## Abstract

While bulk PuO_2_ is known to be strongly resistant to dissolution, even under ultrasonic irradiation, this study demonstrates that nanometric PuO_2_ samples can exhibit enhanced reactivity when sonicated under an Ar/(20 %)O_2_ atmosphere. Sonication of powdered PuO_2_ nanoparticles (∼5 nm) in pure water was found to be ineffective. In contrast, colloidal PuO_2_ nanoparticles (∼3 nm) prepared via hydrolysis exhibited markedly different behavior, leading to the accumulation of Pu(VI), with sonochemically-generated H_2_O_2_ playing a crucial role in the process. Further investigations identified an intermediate species implicated in the dissolution process, agreeing with a recently described Pu(IV) peroxide compound. Despite the chemical similarity of the PuO_2_ nanoparticles, this study highlights their dual reactivity under conditions favoring H_2_O_2_ formation highlighting an important role of the material’s preparation method. Beyond underscoring the critical role of H_2_O_2_ in the reactivity of PuO_2_ nanoparticles, this study also evidences a potential pathway for their transformation under environmental conditions where radiolysis can generate similar chemical environments.

## 1 Introduction

Plutonium is a byproduct of uranium fission in nuclear reactors and plays an important role in both nuclear weapons production and nuclear energy. In the latter, the element plutonium is used in the preparation of mixed oxide (MOX) fuel in the form of plutonium dioxide (PuO_2_) [[1], [2], [3], [4]]. PuO_2_ crystallizes in the fluorite structure of the face-centered cubic system (Fm-3 m space group) and is distinguished by its high thermodynamic stability and very poorly soluble nature. It is well established that PuO_2_ is thermodynamically insoluble in acidic and non-complexing media, as evidenced by its positive free energy (Δ_r_G°= +32 kJ.mol^−1^) [5,6]. The most applied method for dissolving PuO_2_ rely on the use of boiling concentrated nitric acid with the addition of dilute hydrofluoric acid [4,[7], [8], [9]]. However, the ability of plutonium to exist in four different oxidation states under aqueous conditions has led to the exploration of alternative dissolution strategies. One such approach is the oxidative dissolution of PuO_2_ in nitric acid, catalyzed by strong oxidants such as Ce(IV), O_3_ or Ag(II), which enable efficient dissolution kinetics at near-room temperature [6,8,[10], [11], [12], [13]]. While these approaches can achieve significant PuO_2_ dissolution, several drawbacks have been consistently reported, including equipment corrosion, excessive reagent consumption, and the hazardous nature of the reaction media. Alternatively, reductive dissolution methods allowed for the investigation of far less aggressive environment [8,12,14,15]. Thermodynamic studies have indicated that the reductive dissolution of PuO_2__(s)_ into Pu(III)_(aq)_ can occur with redox couples exhibiting a potential lower than approximately 0.5 V vs. SHE (Δ_r_G° = −65 kJ mol^−1^). Various metal ions reported in the literature, including Eu^2+^, V^2+^, Ti^3+^, Ti^2+^, Cr^2+^, and U^4+^, provide alternatives to conventional approaches while ensuring efficient conversion rates and favorable reaction kinetics under mild conditions.

Sonochemistry has emerged as a promising tool in actinide chemistry, both in homogeneous solutions and heterogeneous solid/liquid systems [[16], [17], [18]]. In aqueous solutions, early studies highlighted the potential for redox control of dissolved plutonium species through sonochemical modifications of the reaction medium. For instance, Dalodière et al. demonstrated that Pu(VI) is selectively reduced to Pu(V) in dilute nitric acid (0.25–1 M) under an Ar/20 % O_2_ gas mixture promoting the in-situ formation of H_2_O_2_ [19]. Similarly, the sonochemical generation of H_2_O_2_ has been shown to play a key role in the redox stabilization of Pu(IV) and Pu(III) in aqueous nitric acid solutions [20]. Moreover, the combined physical effects of cavitation, along with the in-situ formation of reactive species, have significantly enhanced the dissolution of materials resistant to dissolution [[21], [22], [23], [24], [25], [26]]. Nikonov and Shilov were the first to report the use of 44 kHz ultrasound to enhance the oxidative dissolution of PuO_2_ in 1 M LiOH solutions under ozone-rich conditions, achieving a significant acceleration compared to silent conditions [27]. Juillet et al. later observed a three- to fourfold increase in PuO_2_ dissolution rates in 4 M nitric acid using direct-contact sonochemical systems at 20 and 500 kHz [28]. The 20 kHz sonochemical dissolution of PuO_2_ was found to be significant in HCl medium in the presence of TiCl_3_ as a source of reducing species [29]. In line with this work, the introduction of metal Ti particles under 20 kHz ultrasonic irradiation enabled the accumulation of Pu(III) in 0.5 M HNO_3_ / 2 M HCOOH / 0.1 M HN (HN = hydrazinium nitrate), providing evidence for the reductive dissolution of PuO_2_ in aqueous nitric conditions (Eq. (1), (2)) [22]. The addition of HF or NH_4_F aliquots in the system further enhanced the dissolution rate by promoting titanium dissolution through fluoride complexation and facilitating the effective interaction of dissolved Ti species with the PuO_2_ surface. More generally, these work also confirmed the increase of the resistance to dissolution of the plutonium oxides with the firing conditions at the preparation stage [1,2,5,6,9,13,14,22,29,30].

(1) Ti_(s)_→)))→Ti^2+/3+^_(aq)_

(2) PuO_2(s)_+Ti^3+^_(aq)_+2H^+^→Pu^3+^_(aq)_+TiO^2+^_(aq)_+H_2_O

The increasing interest in nanomaterials has driven recent studies to explore the preparation, characterization, and reactivity of nanosized actinide oxides including nanoparticles (NPs), colloids or nanostructured oxides [21,[31], [32], [33], [34], [35], [36], [37], [38], [39], [40]]. In particular, the sonication of ThO_2_ and UO_2_, which crystallize isostructurally with PuO_2_, has revealed higher reactivity under ultrasonic irradiation in comparison to bulk counterparts [21,23]. Bonato et al. investigated the sonochemical behavior of nanostructured and nanopowdered ThO_2_ samples in dilute sulfuric acid solutions and highlighted the substantial dissolution of the samples along with the formation of secondary phases later identified as thorium peroxosulfates [21,41]. In this system, a combined influence of the acoustic cavitation, in-situ generated H_2_O_2_, and sulfuric acid concentration was demonstrated. More recently, the sonication of UO_2_ samples in pure water and aqueous sulfuric acid solutions under an Ar/(20 %)O_2_ atmosphere revealed the complete transformation of the initial oxide into uranyl peroxides. Such transformation involved the oxidative dissolution of U(IV)_s_ into U(VI)_aq_ and the reprecipitation of the latter with the in-situ formed hydrogen peroxide [23]. A careful selection of the experimental conditions allowed an original pseudomorphic transformation of the UO_2_ micrometric platelets, which exhibited an additional through-hole extending across the entire sample for specific conditions. Such a phenomenon was attributed to a sonocapillary effect, which facilitated the penetration of reactants into the nanostructured UO_2_ samples.

Based on these observations, the in-situ generation of H_2_O_2_, combined with the specific physical effects induced by acoustic cavitation, led to characteristic transformations in ThO_2_ and UO_2_ samples, challenging potential reactivity with other actinide oxides. The current study investigates the sonochemical behavior of PuO_2_ NPs in pure water under an Ar/(20 %)O_2_ atmosphere providing an increased and in-situ generation of H_2_O_2_. Two synthesis approaches were considered for the preparation of the PuO_2_ NPs: (i) a powdered route involving solution precipitation with ammonia followed by calcination, and (ii) an aqueous route based on the hydrolysis of Pu(IV) in pure water leading to a stabilized colloidal suspension. The response of these materials to ultrasonic irradiation, in conditions favoring the enhanced formation of H_2_O_2_, was monitored and is discussed in this paper.

## 2 Experimental section

***Caution!*** *^239^Pu is an α-emitting radioisotope and standard precautions should be followed for handling this chemical element.*

### 2.1 Syntheses

A concentrated Pu(IV) solution was prepared by purification onto anion exchange resin before stabilization in *ca.* 2.4 M HNO_3_. The isotopic composition (wt%) was 96.9 % ^239^Pu, 2.9 % ^240^Pu, 0.1 % ^242^Pu and 0.1 % ^238^Pu. All of the other used reagents were of analytical grade and supplied by Sigma-Aldrich. The aqueous solutions were prepared using deionized water (18.2 MΩ.cm at 25 °C). Two nanoscaled PuO_2_ samples were considered: (i) PuO_2_ nanopowder was obtained in basic aqueous conditions in the presence of polyethylene glycol (PEG, M = 3000 g.mol^−1^, 2.5 wt%) [31,42]. Briefly, an ammonia solution (30 wt%) was slowly added to a Pu(IV) solution in the presence of PEG until the pH reached a value of 10. One hour after mixing the solutions, the as-obtained Pu precipitate was separated from the supernatant by centrifugation, washed twice with water and dried at room temperature under the atmosphere (air circulation) of the glovebox. Then, the sample was calcined under air at 485 °C (2 h). (ii) PuO_2_ colloidal NPs were prepared by diluting a Pu(IV) solution aliquot, previously stabilized in a nitric medium, into pure water under mechanical stirring. Typically, a 1 mM colloidal suspension can be prepared by diluting 87 µL of 0.575 M Pu(IV) stabilized in 2.4 M HNO_3_ solution into 50 mL of pure H_2_O (18.2 MΩ.cm at 25 °C) [43,44]. After *ca.* 1 h, the formation of PuO_2_ colloids was indicated by the typical green color of the solution and its electronic signature characterized with visible absorption bands located at 578, 616, 688 and 735 nm. The additional presence of traces of Pu(VI) observed at 830 nm was attributed to the disproportionation reaction of Pu(IV) that competes with the colloid formation during hydrolysis [45].

Table 1 summarizes the characteristics of the as-prepared PuO_2_ samples. Both samples are composed of quasi-spherical NPs of nearly the same size, about 3 nm and 5 nm for the colloidal and powdered samples, respectively. The crystalline nature of both samples, consistent with PuO_2_ involving Pu exclusively at the +(IV) oxidation state, has been previously evidenced using XRD and EXAFS spectroscopy. HR-TEM pictures are provided in Fig. S1, SI [31,43,44]. Finally, the main difference between these two samples is that one is in the colloidal state stabilized in an aqueous solution, while the other has undergone thermal treatment in the powder form.

**Table 1:** Characteristics of the nanosized PuO_2_ samples used in this study [31,43,44].

**PuO_2_ Sample** Preparation conditions	**PuO_2_ colloidal NPs** Hydrolysis route (20 °C, 1 month)		**PuO_2_ nanopowder** Basic route (485 °C, 2 h)	
**TEM morphology**	Quasi-spherical, dispersed NPs		Quasi-spherical, agglomerated NPs	
**S_BET_ (m^2^.g^−1^)**	180*		79 ± 1	
**Particle size** **(nm)**	*HR-TEM*	*SAXS*	*HR-TEM*	XRD**
	2.9 ± 0.9	2.6 ± 0.9	4.6 ± 1.0	5.1 ± 0.1

*Calculated from geometric HR-TEM parameters and density,

** Rietveld refinement.

### 2.2 Sonochemical experiments

Sonochemical experiments were performed in Atalante facility (Marcoule, France) in a dedicated glovebox in the presence of PuO_2_ colloidal solutions (1 – 10 mM, 0.2–2.4 g.L^-1^) or powdered PuO_2_ NPs (15 mM, 3.5 g.L^-1^) dispersed in pure water.

The experiments at low frequency ultrasound (20 kHz) were performed in a thermostated batch reactor containing 50 mL of solution with a 1 cm^2^ titanium alloy probe (P_ac_ = 0.34 W.mL^−1^) fitted on top of the reactor and connected to a 750 W generator (Sonics & materials, Vibracell VCX 750). The high-frequency ultrasound experiments (205 kHz) were conducted in a thermostatically controlled cylindrical reactor with a volume of 250 mL (P_ac_ = 0.13 W.mL^−1^). The reactor was equipped with a 25 cm^2^ irradiating surface (ELAC Nautik) connected to a 125 W multi-frequency generator. To ensure uniform suspension and prevent the formation of standing waves, an additional mechanical stirring was provided with a glass blade (100 rpm) for high-frequency experiments.

For all the experiments, the solutions were sparged with Ar/(20 %)O_2_ (Air Liquide, 99.8 %) about 20 min before sonication and during the whole ultrasonic treatment at a controlled rate of 100 mL.min^−1^. The temperature inside the reactor during the sonolysis was maintained at 20 °C with a cryostat (Lauda proline) placed outside the glovebox and connected to the reactor. The temperature was measured by a thermocouple immersed into the solution. The acoustic power density (W.mL^−1^) transmitted to the solution was measured using the conventional thermal probe method [18].

### 2.3 Analyses and characterizations

#### 2.3.1 UV–Vis absorption spectroscopy

The concentration of Pu in solution was followed by UV–Vis absorption spectroscopy. During sonolysis, aliquots of solutions (1 mL) were collected with a syringe through a septum and filtered using 0.2 µm PTFE filters. Absorption spectra were recorded in a 1 cm quartz cuvette from 350 to 850 nm using a Shimadzu UV3600 spectrophotometer connected to the glovebox via optical fibers. The Pu concentration was followed by using the molar absorption coefficient associated to the main absorption bands of the respective oxidation states provided in Table 2. The statistical error was estimated to be lower than 10 %.

**Table 2:** Parameters used to determine the Pu concentration with visible absorption spectroscopy [20,46,47].

**Pu oxidation state**	**Main absorption band** **(nm)**	**Molar coefficient** **(cm^−1^.M^−1^)**
III	602	34
IV	476	70
VI	830	470

#### 2.3.2 Powder X-ray diffraction (PXRD)

PXRD diagrams were obtained with the use of a Bruker D8 Advance X-ray diffractometer equipped with a linear Lynx-eye detector (Cu Kα_1,2_ radiation, λ = 1.54184 Å). PuO_2_ samples were immobilized in epoxy resin to prevent the dispersal of harmful radioactive dust. The addition of gold as an internal standard was also carried out (JCPDS 00–004-0784). PXRD patterns were recorded between 10° and 80° (θ-2θ mode) at room temperature, with a step size of Δ(2θ) = 0.01°. Powder X-Ray Diffraction (PXRD) confirmed the characteristic patterns of the fluorite structure (Fm-3 m space group) typical for PuO_2_ in agreement with the literature and our previous report [31,48].

#### 2.3.3 Experiments with synchrotron radiations

Small-Angle X-ray Scattering (SAXS) experiments were performed on the MARS beamline at the synchrotron SOLEIL, with the main experimental parameters provided in Table 3. These experiments allowed for the investigation of the size and shape of the colloidal NPs, offering insights into their structural evolution under different conditions. X-ray Absorption Spectroscopy (XAS) was conducted using a double-crystal Si[111] monochromator and a 13-element Ge detector. Energy calibration was performed using a Zr foil (edge at 17998 eV) as a reference for Pu. Measurements were performed by integrating the L_α1_ fluorescence signal at the Pu L_3_-edge, enabling the determination of both the oxidation state and local structure of Pu atoms. The EXAFS signal processing is based on the simulation of oscillations showing interactions between the Pu-O and Pu-Pu coordination spheres, calculated using the FEFF 8.4 software from the crystallographic structure of PuO_2_. These oscillations were then fitted to reproduce the experimental data by adjusting bond lengths, coordination numbers, and Debye-Waller parameters, using the Athena and Artemis software with IFFEFIT extensions [49]. This methodology enabled a precise determination of the local atomic environment, shedding light on potential structural modifications or phase transitions induced by experimental conditions.

**Table 3:** Main experimental parameters for SAXS measurements conducted on the MARS beamline (SOLEIL), where e_sample_ is the sample thickness, d is the sample-to-detector distance, and t_sample_ is the exposure time.

**λ beam (nm)**	**detector**	**e**_sample_**(cm)**	**d (cm)**	**t_sample_ (s)**
0.7294	Pilatus	0.52	76.2	2–5

## 3 Results and discussion

### 3.1 Sonochemical behavior of PuO_2_ NPs in pure water: Powdered (basic route) vs colloidal (hydrolysis route) samples

**Fig. 1:**
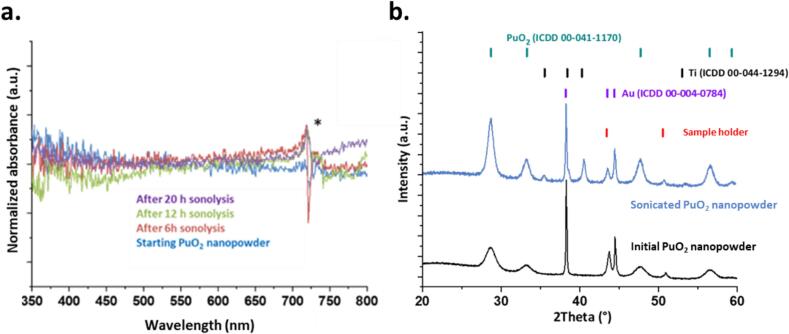
(a) Vis. absorption spectra acquired on filtered aliquots obtained during the sonication of powdered PuO_2_ NPs at 20 kHz (20 °C, Ar/(20 %)O_2_, 0.34 W.mL^−1^). (b) XRD patterns acquired on the PuO_2_ powder before and after sonication. *Artefact from the optical fibers.

The absorption spectra acquired during the sonication of powdered PuO_2_ nanoparticles (∼5 nm) in pure water under an Ar/(20 %)O_2_ atmosphere confirmed the hardly-soluble nature of this oxide toward dissolution (Fig. 1a). No absorption bands associated with Pu species in solution were observed. Moreover, the addition of AgO to the supernatant of the centrifuged solution, intended to oxidize potential Pu(IV) species into Pu(VI) for easier identification (due to its molar coefficient), was inconclusive. The X-ray diffraction (XRD) patterns of the solid residues obtained after ultrasonic irradiation of the powdered NPs in pure H_2_O exhibited the characteristic diffraction peaks of PuO_2_, which crystallizes in a face-centered cubic structure (space group Fm-3 m), in agreement with both the reference pattern (JCPDS 00-041-1170) and the untreated sample [31,48]. Additional diffraction peaks corresponding to titanium (Ti, JCPDS 00-044-1294) were also observed and were attributed to the accumulation of Ti particles due to the erosion of the ultrasonic probe during the experiment. Overall, these analyses confirmed the lack of reactivity of powdered PuO_2_ nanoparticles under these conditions and the absence of secondary phases formed during sonication.

**Fig. 2:**
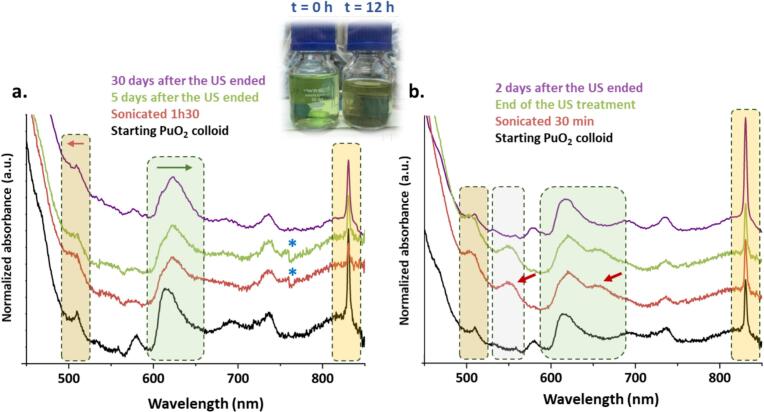
Evolution of the visible absorption spectra observed during the sonication of a PuO_2_ colloidal suspension (Ar/(20 %)O_2_, 20 °C, pH = 2) at (a) 20 kHz (0.34 W.mL^−1^) and (b) 205 kHz (0.13 W.mL^−1^). Inset: photograph of the colloidal PuO_2_ solution before and after 12 h sonolysis. *Artefact from the optical fibers.

Under similar experimental conditions, the sonication of a colloidal suspension of PuO_2_ NPs (∼3 nm), prepared by hydrolysis of Pu(IV) stabilized in acidic nitric conditions, contrasted with the results previously described for powdered PuO_2_ NPs. At this stage, it is important to recall that the colloidal NPs are dispersed and stabilized in their synthesis medium, which essentially corresponds to an aqueous acidic solution (pH = 2) containing nitrates originating from the Pu mother solution. The Fig. 2a gathers the visible absorption spectra acquired during the 20 kHz sonication of a 1 mM colloidal suspension of PuO_2_ NPs at 20 °C under Ar/(20 %)O_2_ atmosphere. Sonication for 1.5 h (red spectrum) lead to a distortion and red shift of the broad absorption band of the PuO_2_ colloid, typically observed near 610–620 nm, along with a shift in the absorption band from 510 nm to 505 nm. This spectrum remained stable and did not show any significant modification 30 days after the experiment (green and purple spectra), except for a slight increase in Pu(VI) concentration observed at 830 nm. A similar experiment was carried out at a higher ultrasonic frequency (205 kHz) to enhance the sonochemical activity of the system (Fig. 2b). This approach, which is expected to increase the formation rate of H_2_O_2_ under ultrasound [21,50,51] led to a darkening of the colloidal suspension (Fig. 2, Insert). After 30 min of treatment, the analysis of the electronic spectra revealed the same wavelength shifts at 510 and 640 nm, along with the emergence of two new absorption bands at 550 and 665 nm (red arrows). Unlike the experiments conducted at 20 kHz, the absorption spectrum of the solution continued to evolve after the ultrasound irradiation stopped. Within 24 h, the spectra returned to that of the initial colloidal suspension, accompanied by a significant increase in the Pu(VI) signature at 830 nm.

**Fig. 3:**
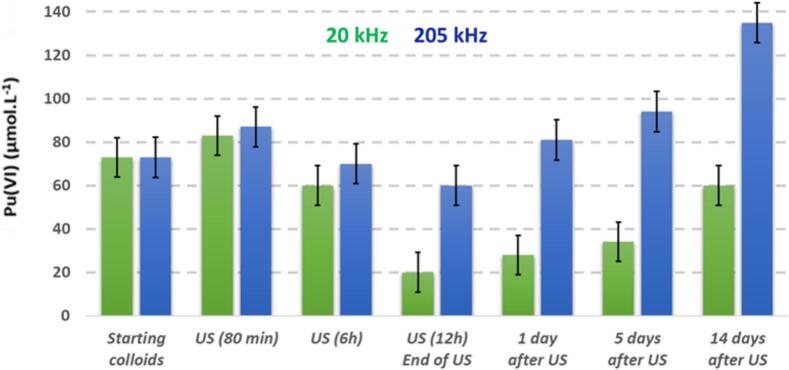
Evolution of Pu(VI) concentration in solution, estimated from its UV–Vis absorption band (λ = 830 nm), during and after sonication of a 1 mM PuO_2_ colloidal solution (Ar/(20 %)O_2_, 20 °C, pH = 2) at (a) 20 kHz (0.34 W·mL^−1^) and (b) 205 kHz (0.13 W·mL^−1^).

Fig. 3 illustrates the evolution of Pu(VI) concentration during and after sonolysis at 20 kHz and 205 kHz. Both ultrasonic frequencies exhibited a similar behavior, characterized by a decrease in Pu(VI) concentration during sonication, followed by a continuous increase over several days after the ultrasound was stopped. The increase in Pu(VI) concentration was more pronounced in the experiment conducted at the higher ultrasonic frequency, eventually exceeding the initial concentration.

Initially, it is important to note the comparable trends observed for Pu(VI) in the experiments conducted at both 20 kHz and 205 kHz. Such behavior excludes, a priori, a significant contribution from Ti particles generated by the erosion of the ultrasonic probe, as this does not occur in high-frequency experiments because this transducer is covered by stainless steel plate. Additionally, previous studies have shown that the sonolysis of Pu(VI) solutions under an Ar/(20 %)O_2_ atmosphere allows for its reduction into Pu(V) with H_2_O_2_ and potential back-oxidation with HO_2_^•^ radicals for low concentrations of H_2_O_2_ (Eq. (4), (5)) [19]. Pu(V) was not detected in our system, likely due to the low Pu(V) concentrations involved and its significantly lower molar absorption coefficient (ε = 19 L·mol^−1^·cm^−1^ at λ = 569 nm, compared to ε = 470 L·mol^−1^·cm^−1^ at λ = 830 nm for Pu(VI)). Taking into account this information, the initial observed variations in Pu(VI) concentration under our experimental conditions, which also appeared to increase at higher frequencies under Ar/(20 %)O_2_ were attributed to the sonochemical formation of H_2_O_2_ and related radical species.

(3) 2PuO_2_^2+^+H_2_O_2_→2PuO_2_^+^+O_2_+2H^+^

(4) PuO_2_^+^+HO_2_^•^+H^+^→PuO_2_^2+^+H_2_O_2_

After sonication, it should be noted that the accumulation of Pu(VI) is preceded by changes in the electronic signature detected by UV–Vis absorption spectroscopy and the disappearance of unknown absorption bands at 550 and 665 nm. This observation suggests that the accumulation of Pu(VI) may be associated with the partial dissolution of PuO_2_ NPs and the possible formation of a transient species at the surface of PuO_2_ NPs in the presence of H_2_O_2_. The latter could facilitate the dissolution of the PuO_2_ NPs and ultimately lead to the accumulation of Pu(VI). Note that the monitoring of H_2_O_2_ concentration, using the classical Ti(IV) method, was not feasible due to the strong absorption of the colloidal suspension in the near-UV region [51]. Although the mechanism involved needs further elucidation, it is worth noting that Pu(IV) desorption from mineral surfaces was also observed at low H_2_O_2_ concentrations [52]. Two hypotheses can be proposed to explain the accumulation of Pu(VI) after the sonolysis of PuO_2_ colloidal NPs in an oxygenated atmosphere:

(i) By analogy with the Fenton-like mechanism proposed by Finkelstein et al. to describe the formation of (meta-)studtite from UO_2_ via UO_2_^+^ (Eq. (5), the first hypothesis involves the oxidation of PuO_2_ by adsorbed H_2_O_2_, leading to the formation of Pu(V) (Eq. (6)) [[53], [54], [55]]. A subsequent oxidation or dismutation step of Pu(V) would accumulate Pu(VI). Note also that a recent study dedicated to PuO_2_ surface radiolysis indicated that, although presumably important, the fate of H_2_O_2_ on the PuO_2_ surface is still unclear. By analogy with the complex set of catalytic reactions occurring with UO_2_ in the presence of H_2_O_2_, the authors indicated that H_2_O_2_ may interact with PuO_2_ surfaces, forming HO^•^ and HO_2_^•^ radicals through a series of reactions (Eq. (8), (9)) [56].

(5) UO_2_+H_2_O_2_→UO_2_^+^+HO^•^+HO^−^

(6) PuO_2_+H_2_O_2_→PuO_2_^+^+HO^•^+HO^−^

(7) PuO_2_+H_2_O_2_→PuO_2_---2HO^•^_(ads)_

(8) PuO_2_--–HO^•^_(ads)_+H_2_O_2_→PuO_2_+HO_2_^•^+H_2_O

(ii) The second hypothesis supports a reductive dissolution mechanism occurring at the surface of PuO_2_ NPs. This hypothesis involves a Pu(IV) peroxide intermediate, analogous to what has been reported in Pu(IV) solution chemistry or electrochemistry and supported by the observed variation in absorption spectroscopy [[57], [58], [59], [60], [61]]. It has been reported that H_2_O_2_ can act as both an oxidizing (E°(H_2_O_2_/H_2_O) = 1.77 V vs. SHE) and reducing (E°(O_2_/H_2_O_2_) = 0.68 V vs. SHE) agent for Pu(IV) and Pu(III), respectively. Additionally, the reduction of Pu(IV) by H_2_O_2_ is known to involve the transient formation of a Pu(IV) peroxide complex which decomposes into Pu(III) in the presence of protons (Eq. (9), (10). In this case, the observed accumulation of Pu(VI) can then result from successive reactions including oxidation of Pu(III) and disproportionation of Pu(IV) ((11), (12)) [45]. In solution chemistry, these redox processes allow considering Pu as a catalyst for H_2_O_2_ decomposition until a Pu(IV)/Pu(III) steady state is reached. A plausible hypothesis is that redox processes at the PuO_2_ NP surface could involve similar peroxide intermediates characterized by the UV–Vis shifts and their decomposition as a function of the medium acidity. Note also that the redox behavior of the Pu(IV)/Pu(III) couple in the presence of sonochemically-generated H_2_O_2_ has already been observed in solution and agreed with the above reported behaviour [20].

(9) 2Pu(IV)+H_2_O_2_+H_2_O→Pu_2_(O_2_)(OH)^5+^+3H^+^

(10) Pu_2_(O_2_)(OH)^5+^+H^+^→2Pu(III)+O_2_+H_2_O

(11) 2Pu(III)+H_2_O_2_+2H^+^→2Pu(IV)+2H_2_O

(12) 3Pu(IV)+2H_2_O⇌2Pu(III)+Pu(VI)+4H^+^

### 3.2 Characterization of the intermediate species

**Fig. 4:**
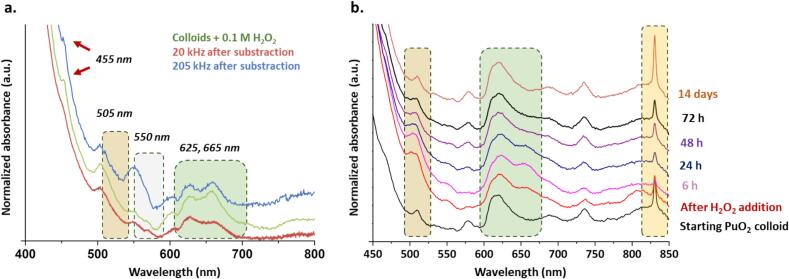
(a) Spectra obtained under ultrasonic irradiation at 20 kHz (red spectrum) and 205 kHz (blue spectrum) of a Pu(IV) colloidal solution (pH_th_ = 1.5). The displayed signals result from the subtraction of the initial contribution. (b) Evolution of a Pu colloidal solution in the presence of 0.1 M H_2_O_2_ (green signal on a. after subtraction).

The UV–Vis spectral contribution of the transient species was isolated by subtracting the spectrum of the PuO_2_ colloid reference from the spectra obtained after 30 min of sonication at 205 kHz and 1.5 h at 20 kHz (Fig. 4a). The resulting spectra revealed distinct spectral signatures for both frequencies, characterized by absorption bands at 505, 550, 600, 625, and 665 nm. A small band at 455 nm was also observed, along with strong absorption in the near-UV range. In agreement with the discussion above, similar spectral signatures were isolated for both low- and high-frequency ultrasound experiments. It is worth noting that the bands at 550 and 600 nm differ from those typically associated with Pu(III) [1,20]. To further investigate the nature of this intermediate species, an additional experiment was conducted by introducing 0.1 M H_2_O_2_ into a 1 mM colloidal solution of PuO_2_ NPs (H_2_O_2_/Pu = 760). The spectroscopy investigations are summarized in the Fig. 4b. Several absorption bands at 505, 550, and 665 nm progressively increased over time under continuous stirring, reaching maximum intensity after approximately 6 h. Subsequently, these absorption bands began to decrease, eventually disappearing after 48 h. Several days later, the initial absorption spectrum of the colloidal PuO_2_ NPs reappeared, accompanied by an increase in Pu(VI) concentration. Such a behavior agrees with our previous sonochemical observations. In addition, the intermediate absorption spectrum obtained after H_2_O_2_ addition revealed strong similarities with those resulting from ultrasonic treatments (green vs. blue and red spectra, Fig. 4a), except for the band at 550 nm in the high-frequency experiment. These results confirmed the contribution of H_2_O_2_ in the formation of these spectral bands concluding in the formation of a Pu-peroxide intermediate in the system.

**Fig. 5:**
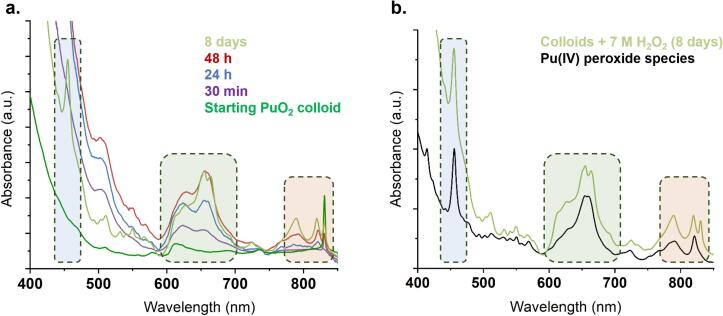
(a) Spectroscopic evolution of the colloidal PuO_2_ NPs (10 mM) in the presence of added 7 M H_2_O_2._ (b) Comparison of the signal observed after 8 days in (a) with the one of the Pu(IV) peroxide species formed by dilution of an acidic Pu(IV) nitric solution into a H_2_O_2_ solution in excess.

Complementary experiments were conducted with aqueous solutions of Pu(IV) to investigate the role of Pu and H_2_O_2_ concentrations in the system (summary provided in Table S1, SI). When the H_2_O_2_ concentration was kept constant while increasing the Pu concentration (thus reducing the H_2_O_2_/Pu ratio), the intermediate species became less stable over time. For instance, in a solution containing 5 mM Pu (H_2_O_2_/Pu = 150), the characteristic 665 nm band nearly disappeared after 24 h, whereas it remained present in the 1 mM Pu system (H_2_O_2_/Pu = 760) (Fig. S2, SI). This behavior was attributed to differences in solution acidities between the two conditions (0.03 mmol of HNO_3_ for 1 mM Pu vs. 0.2 mmol for 5 mM Pu). This behavior agrees with a possible decomposition of the species more rapidly as acidity increases as already reported for Pu(IV) peroxide complexes [61,62]. For higher concentrations (7 M H_2_O_2_, 10 mM Pu), well-defined spectra characterized with the previously observed absorption bands were recorded. The 455 and 655 nm bands were found to increase over time (Fig. 5a) in addition to the increase of new bands at 790 and 821 nm. It is worth noting that this last signature differs with the postulated peroxide dimers of typical “brown” and “red” colors described in the literature [57,60,61,63]. By contrast, this signature agrees with a recently described Pu(IV) peroxide compound of green color observed when diluting Pu(IV) aliquots into large volumes of H_2_O_2_ solutions [64]. A comparison of the spectra acquired in the present study with this peroxide compound is provided in Fig. 5b confirming the formation of the latter in our conditions for the highest concentration of Pu and H_2_O_2_.

**Fig. 6:**
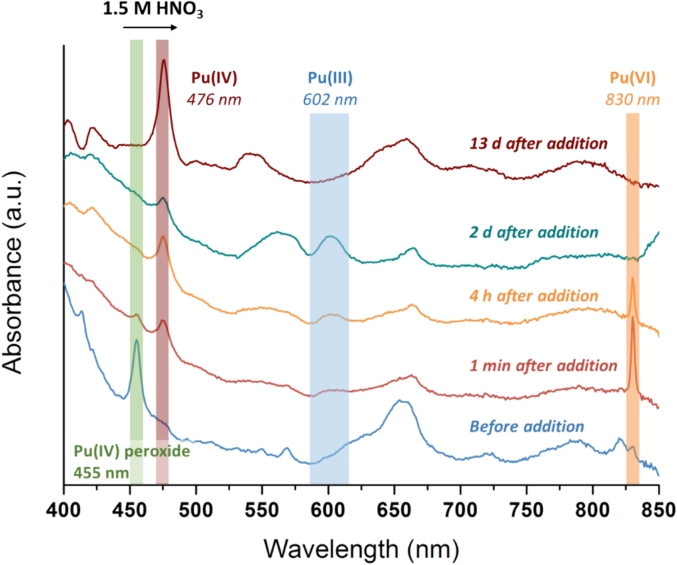
Evolution of the Pu(IV) green peroxide in the presence of 1.5 M HNO_3_ (absorption spectroscopy). The Pu(IV) peroxide species was formed by diluting acidic Pu(IV) into a H_2_O_2_ solution.

The exact nature of this green peroxo compound remains speculative at this stage, and its formation and growth are not yet well established. The absence of certain absorption bands at the lowest concentrations studied (particularly in the sample formed under ultrasound irradiation) can be attributed to the low abundance of the species and possibly to the presence of Pu green peroxo precursors before polymerization. Nevertheless, previous studies demonstrated the strong stability of the Pu(IV) peroxide compound in slightly acidic solutions. By contrast, acidic conditions (1.5 M HNO_3_) were found to promote its decomposition and conversion into Pu(III) and Pu(VI), and ultimately into Pu(IV) (Fig. 6). This observation confirmed the mechanism dealing with the formation of an intermediate peroxide compound at the PuO_2_ surface followed by its decomposition in analogy with Pu(IV) solution chemistry [57,[61], [62], [63]].

**Fig. 7:**
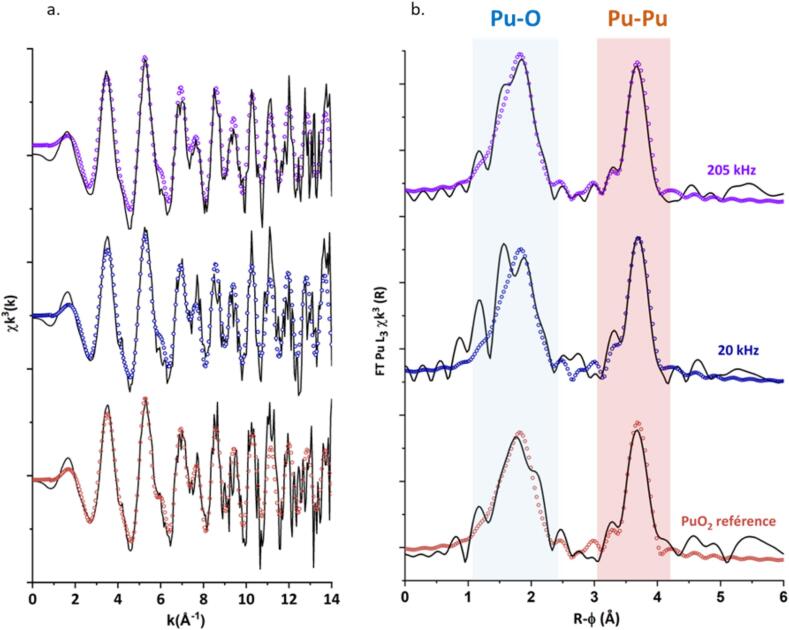
(a) k^3^-weighted EXAFS spectra at the Pu L_3_ edge. (b) Corresponding FT and simulations (circles) in the 2–14 Å^-1^ range for an untreated PuO_2_ colloid solution (reference, red) and the sonicated ones at 20 kHz (12 h, blue) and at 205 kHz (12 h, purple).

Complementary characterizations performed using the synchrotron facility (MARS, SOLEIL) provided normalized Small-Angle X-ray Scattering (SAXS) patterns for colloidal PuO_2_ NP solutions after 20 and 205 kHz sonochemical treatments (Fig. S3, SI). A significant difference was observed both between the scattering profiles of each sample and compared to the initial scattering profile of PuO_2_ colloid reported by Cot-Auriol et al. and Micheau et al. [44,65] Although they retained their colloidal nature even after ultrasonic treatment, the SAXS profiles evidenced significant variations in both the size and the morphology for the PuO_2_ NPs. However, due to a lack of further evidence, we refrained from extracting quantitative information from these signals. X-ray absorption spectroscopy confirmed that Pu predominantly adopted the +(IV) oxidation state in the sonicated systems, with a white line observed at 18,067.2 ± 0.5 eV, consistent with untreated PuO_2_ colloidal NPs (Fig. S4, SI) [[31], [32], [33]]. The absence of a pronounced “yl” shoulder in the XANES spectra confirmed that Pu(VI) remained a minor species (7 % and 14 % at 20 and 205 kHz, respectively) [66]. The EXAFS oscillations and corresponding Fourier transforms (FT) are provided in Fig. 7. The latter is a pseudo-radial distribution that corresponds to the atomic distribution of atoms in the plutonium coordination sphere. The two main signals are attributed to the Pu–O and Pu–Pu coordination spheres, typical of PuO_2_ [20,[31], [32], [33],^43^,^48^,^67^]. The structural parameters obtained from the fit (Table 4) show Pu–O and Pu–Pu distances of 2.31(1) and 3.80(1) Å, respectively, in good agreement with the crystalline nature of bulk PuO_2_.

**Table 4:** Structural parameters determined by EXAFS for PuO_2_ colloidal NPs before and after ultrasonic treatment (20 kHz and 205 kHz). R-factor: 8 %, S_0_^2^ = 1.0, CN: coordination number, σ^2^: Debye-Waller factor, ΔE_0_ = 4.8 eV.

**Sample**		**CN**	**σ^2^ (Å^2^)**	**R (Å)**
**PuO_2_ ref.**	Pu-O	8.0*	0.015 (0.002)	2.31 (0.01)
	Pu-Pu	4.9 (0.9)	0.006 (0.002)	3.80 (0.01)
**20 kHz**	Pu-O	8.0*	0,015 (0,002)	2.32 (0.01)
	Pu-Pu	3.8 (1.4)	0.005 (0.002)	3.81 (0.01)
**205 kHz**	Pu-O	8.0*	0,012 (0,002)	2.32 (0.01)
	Pu-Pu	4.7 (1.0)	0.005 (0.002)	3.81 (0.01)

* Fixed parameters.

Compared to bulk PuO_2_, the extracted data aligned well with PuO_2_ NPs, which are known to exhibit reduced peak amplitude due to NP size and surface effects [31,33,44]. The strong Debye–Waller factor associated with the Pu–O shell has been attributed to the complex surface chemistry of the NPs and the related structural disorder it creates. Notably, the presence of Pu(VI) in solution is known to contribute to subtle modifications in the Pu–O shell, possibly influencing the EXAFS spectra. The coordination number of the Pu–Pu shell is significantly reduced relative to bulk PuO_2_ (CN = 12) due to the smaller NP size [20,33,44]. Overall, despite these minor spectral variations, the data indicate that the signal of the PuO_2_ NPs remains overwhelmingly predominant in the sonicated sample, in agreement with the untreated one. No signal attributed to a transient species (in particular with Pu-Pu distances different from that of PuO_2_) was observed under these conditions. This finding was attributed to the intermediate instability and low concentration.

### 3.3 Discussion

Based on these results, a key takeaway from this study is the crucial role of H_2_O_2_ in this reaction system. It was demonstrated that the introduction of H_2_O_2_, whether generated sonochemically or added manually, leads to the formation of an intermediate species preceding the dissolution of PuO_2_ NPs and the accumulation of Pu(VI) in solution. The nature of this species is closely related to a Pu(IV) peroxide compound recently described in the literature. Given that PuO_2_ is a hardly-soluble material with respect to dissolution, this observation is particularly significant for understanding both the dissolution behavior and reactivity of PuO_2_ nanoparticles in various environments. Beyond this observation, this work highlighted a stark contrast in reactivity between the colloidal and powdered PuO_2_ NPs. Despite structural similarities, the colloidal PuO_2_ NPs exhibited significant reactivity, leading to the formation of a transient Pu(IV) peroxo compound, whereas the powdered PuO_2_ NPs showed no detectable reactivity, even after extended sonication.

Recently, Plakhova et al. investigated the solubility of 2 nm and 8 nm CeO_2_ NPs synthesized by different techniques and found that their dissolution in 0.01 M NaClO_4_ did not follow traditional size-dependent models [35,68]. Instead, thermal post-treatment of the NPs at 40 °C resulted in striking solubility differences compared to undried ones with nearly two orders of magnitude at pH 2. The decreased solubility of the dried NPs was attributed to surface state modifications rather than changes in size or crystalline structure, which remained unchanged upon drying. The alteration of surface hydration was suggested as the primary factor affecting the solubility and overall reactivity of the CeO_2_ NPs [68].

This study indicates that the potential dissolution of colloidal PuO_2_ NPs can also be influenced by surface effects. Rather than being primarily governed by particle size, PuO_2_ NP dissolution may be driven by subtle changes in surface chemistry, particularly hydration state. EXAFS investigations have previously indicated that PuO_2_ NPs prepared via hydrolysis exhibit surface differences attributed to an increase concentration of hydroxyl groups at surface in comparison to bulk samples [43,69]. EXAFS and SAXS analyses performed in this study confirmed that the fluorite core structure of the NPs remained largely intact supporting that the observed changes were confined to subtle surface transformations. It is worth noting that surface hydration has been shown to play a critical role in stabilizing nanoparticle interfaces, reducing surface energy by approximately 20–30 % [68]. The surface state of the PuO_2_ NPs appears to be intricately linked to their synthesis method, reinforcing the broader principle that surface processes, rather than particle size alone, are the primary determinants of reactivity in low-solubility oxides. However, in the present work, the difference in nanoparticle size is too significant to be able to categorically state that the surface plays an exclusive role in the observed reactivity. Further comparisons using similar particle sizes with different surface treatments will be helpful in confirming these assumptions.

Although the results obtained on the dissolution of PuO_2_ NPs are particularly promising for the treatment of PuO_2_ colloidal nanoparticles in industrial processes, the central role of hydrogen peroxide (H_2_O_2_) raises important questions regarding its potential interactions with hot particles in the environment [70,71]. It has been well established that H_2_O_2_ plays a predominant role in the oxidative dissolution of UO_2_, promoting the formation of thermodynamically stable polynuclear species capable of migrating over long distances [72,73]. In the case of PuO_2_ nanoparticles, our observations highlight the contribution of a recently described Pu(IV) peroxide compound, which has also been shown to be remarkably stable in aqueous solution [64]. More generally, the current findings have critical implications for nuclear waste management and environmental implications, as H_2_O_2_ has been reported to be present in water as a result of radiolysis or natural processes [52], challenging current assumptions about the long-term immobility of PuO_2_ in aqueous media.

## 4 Conclusion

This study highlights the crucial role of H_2_O_2_ in the reactivity of PuO_2_ NPs. While powdered PuO_2_ NPs remained unreactive under ultrasonic irradiation, colloidal ones prepared via hydrolysis exhibited significant reactivity, leading to the formation of an intermediate Pu(IV) peroxo species preceding Pu(VI) accumulation. Beyond the previously unrecognized pathway for PuO_2_ NPs dissolution, this work revealed that their behavior in aqueous solution is strongly influenced by their preparation method. Although the observed differences in reactivity support a predominant role of the surface state rather than particle size, further investigations are needed to confirm this hypothesis. This work aligns with previous studies on oxide nanoparticles where surface hydration was shown to influence solubility. It also emphasizes the importance of the materials preparation conditions and history that can lead to significant deviations from anticipated behavior based solely on size effects. Given the stability of PuO_2_ in most conditions, the observed interaction with H_2_O_2_ challenges our knowledge about its long-term behavior, particularly in environments where radiolysis or other natural processes generate peroxide species. This observation has critical implications for nuclear waste management, environmental safety, and industrial processes, underscoring the need for further research on actinide behavior in complex and dynamic aqueous systems in the presence of H_2_O_2_.

## CRediT authorship contribution statement

**Julien Margate:** Writing – review & editing, Writing – original draft, Investigation, Data curation. **Matthieu Virot:** Writing – review & editing, Writing – original draft, Validation, Supervision, Methodology, Investigation, Funding acquisition, Data curation, Conceptualization. **Thomas Dumas:** Writing – review & editing, Supervision, Methodology, Data curation. **Simon Bayle:** Writing – review & editing, Data curation. **Denis Menut:** Methodology, Investigation, Data curation. **Laura Bonato:** Writing – original draft, Investigation, Data curation. **Emilie Broussard:** Investigation. **Fanny Molière:** Investigation. **Charles Hours:** Investigation. **Laurent Venault:** Investigation. **Sergey I. Nikitenko:** Writing – review & editing, Supervision, Methodology.

## Declaration of competing interest

The authors declare that they have no known competing financial interests or personal relationships that could have appeared to influence the work reported in this paper.
