# Supplementary material for: Unveiling the dual reactivity of nanoscaled PuO2 sonicated in oxygenated aqueous solutions
Source: Ultrason Sonochem. 2025 Apr 8;117:107346. doi: 10.1016/j.ultsonch.2025.107346 (PMC12017934; doi:10.1016/j.ultsonch.2025.107346)
Supplement: Supplementary Data 1 [file mmc1.docx]

**SUPPORTING INFORMATION**

**Unveiling the Dual Reactivity of Nanoscaled PuO₂ Sonicated in
Oxygenated Aqueous Solutions**

Julien Margate^a^, Matthieu Virot^a^*, Thomas Dumas^b^, Simon Bayle^b^, Denis Menut^c^, Laura Bonato^a^, Emilie Broussard^b^, Fanny Molière^b^, Charles Hours^a^, Laurent Venault^b^, Sergey I. Nikitenko^a^

*^a^ICSM, Univ Montpellier, CEA, CNRS, ENSCM, Marcoule, France*

*^b^CEA, DES, ISEC, DMRC, Univ Montpellier, Marcoule, France*

*^c^Synchrotron SOLEIL, L’Orme des Merisiers, Saint-Aubin, France*

[**matthieu.virot@cea.fr*](mailto:*matthieu.virot@cea.fr)

**Figure S1:** HR-TEM pictures of PuO_2_ colloids and nanopowder. Reproduced from refs [1] and [2]


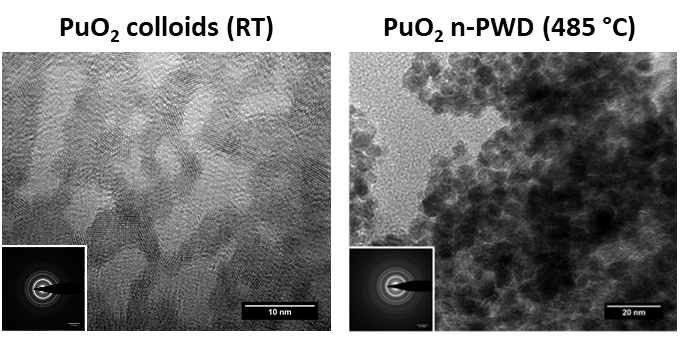


**Figure S2:** Evolution of Vis-NIR absorption spectra of PuO₂ colloidal solutions (1, 5, and 10 mM) in pure water after the addition of H₂O₂ (0.1 M) under the conditions described in Table S1.


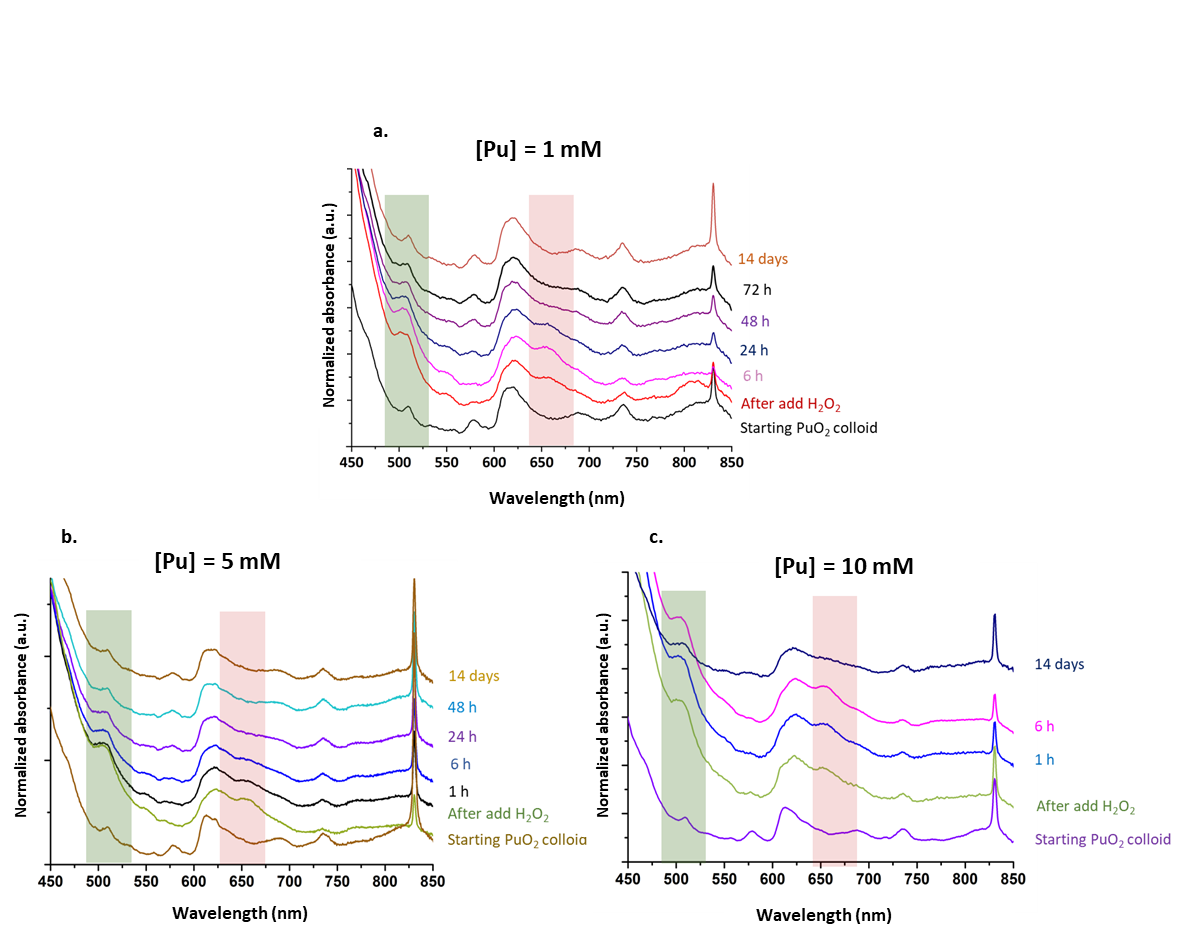


**Table S1:** Operating conditions for the treatment of Pu colloids with H₂O₂.

| **Lot** | **Pu**  **(mM)** | **HNO_3_**  **(mmol)** | **Pu**  **(mmol)** | **H_2_O_2_**  **(M)** | **H_2_O_2_**  **(mmol)** | **H_2_O_2_/Pu** |
| --- | --- | --- | --- | --- | --- | --- |
| **1** | 1 | 0.03 | 5.0 10^-3^ | 0.1 (200 µL) | 3.8 | 760 |
| **2** | 5 | 0.2 | 2.5 10^-2^ | 0.1 (200 µL) | 3.8 | 150 |
| **3** | 10 | 215 | 5.0 10^-2^ | 0.1 (300 µL) | 5.6 | 112 |
| **4** | 10 | 215 | 5.0 10^-2^ | 7 (300 µL) | 400.0 | 8000 |

**Figure S3:** SAXS diagrams acquired on the Pu(IV) colloids after ultrasonic treatment at 20 kHz (green curve) and 205 kHz (red curve) at 1 mM PuO_2_. Lines indicate the curve slopes.


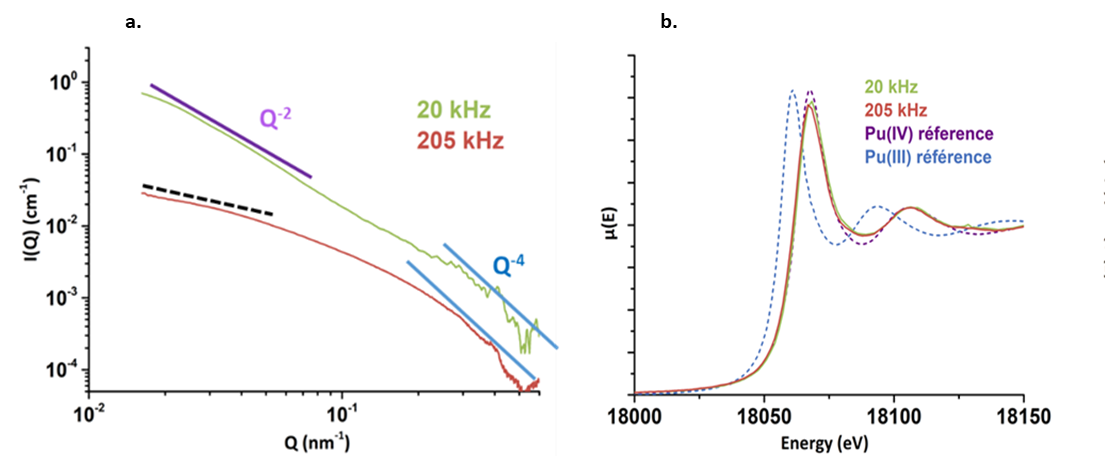


Figure S3:

For the solution treated at 205 kHz, the scattering profile shows a slope trending towards Q^0^ at low scattering vector (Q) values, although it does not fully reach the plateau. At higher Q values, the slope transitions to Q⁻⁴ (red signal). This behavior suggests three-dimensional (3D) particles, which are often spherical and compact in nature. For the solution treated at 20 kHz, a Q⁻⁴ slope is also observed at higher Q values, but at low Q, the slope trends towards Q^-2^ (green signal). This scattering pattern resembles those observed for sonochemically synthesized PuO₂ colloids, where the presence of lamellar or two-dimensional (2D) structures has been proposed.[3,4] Neither scattering profile reaches the Q^0^ plateau, making it difficult to extract quantitative information regarding the arrangement of these species in solution.

**Figure S4:** (a) XANES spectra measured at the Pu L_3_ edge after 20 and 205 kHz sonication of the PuO_2_ colloidal nanoparticles (20 kHz or 203 kHz, 20°C) compared to Pu(III), Pu(IV) and Pu(VI). (b) First derivatives of some XANES spectra.


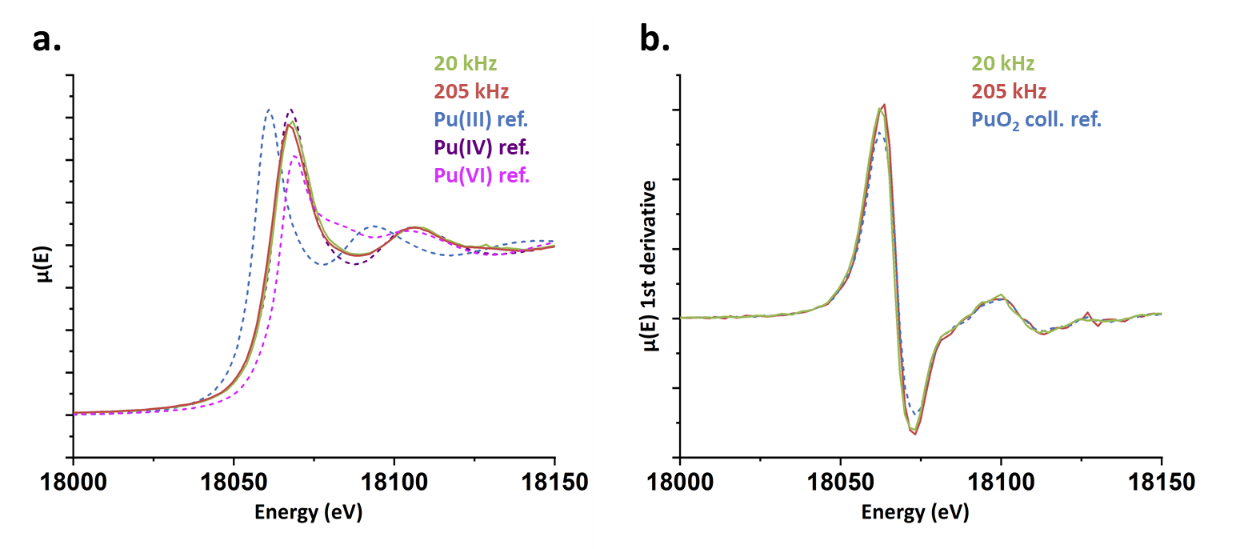


Both Pu(III) and Pu(VI) references correspond to ionic species present in solution as aqua ions, while the Pu(IV) reference was obtained in nitric media. A significant amount of Pu(III) would have induced noticeable shifts in the absorption edges of the samples. In contrast, the contribution of Pu(VI) is more challenging to identify. The slight shift in the white line position, along with the attenuation of the shoulder observed in the sonicated samples, could suggest a minor presence of Pu(VI) in solution, consistent with UV-Vis spectroscopy. However, this interpretation remains uncertain and may be influenced by other phenomena. Therefore, we prefer to adopt a cautious approach and conclude that Pu(IV) is strongly predominant in the studied samples. Further insights into the contributions of different oxidation states would require additional experiments, such as HERFD-XANES measurements at the M₄ edge.[5]

[1] E. Dalodière, M. Virot, V. Morosini, T. Chave, T. Dumas, C. Hennig, T. Wiss, O. Dieste Blanco, D.K. Shuh, T. Tyliszcak, L. Venault, P. Moisy, S.I. Nikitenko, Insights into the sonochemical synthesis and properties of salt-free intrinsic plutonium colloids, Sci. Rep. 7 (2017) 43514. <https://doi.org/10.1038/srep43514>.

[2] L. Bonato, M. Virot, T. Dumas, A. Mesbah, E. Dalodière, O. Dieste Blanco, T. Wiss, X. Le Goff, M. Odorico, D. Prieur, A. Rossberg, L. Venault, N. Dacheux, P. Moisy, S.I. Nikitenko, Probing the local structure of nanoscale actinide oxides: a comparison between PuO_2_ and ThO_2_ nanoparticles rules out PuO_2+x_ hypothesis, Nanoscale Adv. 2 (2020) 214–224.

[3] C. Micheau, M. Virot, S. Dourdain, T. Dumas, D. Menut, P.L. Solari, L. Venault, O. Diat, P. Moisy, S.I. Nikitenko, Relevance of formation conditions to the size, morphology and local structure of intrinsic plutonium colloids, Environ. Sci. Nano 7 (2020) 2252–2266. https://doi.org/10.1039/D0EN00457J.

[4] M. Cot-Auriol, M. Virot, T. Dumas, O. Diat, D. Menut, P. Moisy, S.I. Nikitenko, First observation of [Pu_6_(OH)_4_O_4_]^12+^ cluster during the hydrolytic formation of PuO_2_ nanoparticles using H/D kinetic isotope effect, Chem. Commun. 58 (2022) 13147–13150. <https://doi.org/10.1039/D2CC04990B>.

[5] E. Gerber, A. Romanchuk, S. Weiss, A. Kuzenkova, M. O. J. Y. Hunault, S. Bauters, A. Egorov, S. M. Butorin, S. N. Kalmykov, K. Kvashnina, To form or not to form: PuO_2_ nanoparticles at acidic pH, Environ. Sci.: Nano, 9 (2022) 1509-1518. <https://doi.org/10.1039/D1EN00666E>.
